# Supply chain finance and innovation efficiency: An empirical analysis based on manufacturing SMEs

**DOI:** 10.1371/journal.pone.0286068

**Published:** 2023-07-14

**Authors:** Qiang Wang, Shichao Yuan, Dragana Ostic, Liujun Pan

**Affiliations:** 1 School of Finance & Economics, Jiangsu University Jingjiang College, Jiangsu, China; 2 School of Finance & Economics, Jiangsu University, Jiangsu, China; University of Almeria, SPAIN

## Abstract

This paper firstly demonstrates the positive and negative effects of supply chain finance on the innovation efficiency of China’s small and medium-sized enterprises (SMEs) in the manufacturing industry from the theoretical point of view. Based on the data of 267 manufacturing companies in China Growth Enterprise Market from 2015 to 2019, the DEA-SBM method was used to measure the comprehensive innovation efficiency of different companies, and it was further decomposed into technological innovation efficiency and organizational innovation efficiency. Afterwards, it conducts an empirical analysis through the double fixed effect model, and explores the difference in the impact of supply chain finance on innovation efficiency in enterprises with different industries and different property rights. The results show that supply chain financial services have a strong positive impact on the comprehensive innovation efficiency, technological innovation efficiency and organizational innovation efficiency of manufacturing SMEs. Further, supply chain finance has the most significant improvement on the technological innovation efficiency of the sample of private traditional enterprises, but it has a significant inhibitory effect on the organizational innovation efficiency of the sample of state-owned high-tech enterprises. Therefore, this paper suggests that the development of supply chain financial services should increase support for traditional manufacturing industries; appropriately tilt resources to private enterprises; improve relevant supply chain financial laws and regulations, establish and improve corresponding institutional arrangements, and encourage state-owned enterprises to participate in market competition.

## 1 Introduction

As the backbone of China’s economy, small and medium-sized enterprises (SMEs) in the manufacturing industry are central to economic innovation and development. However, to achieve innovation-driven development, China needs to improve its innovation efficiency. The scale characteristics of small and medium-sized manufacturing enterprises indicates that their innovation efficiency may be higher than that of large enterprises (Mark, 2004 [1]). Yet, for a long time, small and medium-sized manufacturing enterprises have often faced the constraints of insufficient investment in innovation—which limits the improvement of innovation efficiency. In recent years, the vigorous development of supply chain finance has provided a possible solution. As a new mode of financing for SMEs, supply chain finance has effectively broadened the financing channels and solved the problem of financing difficulties, providing a new financing approach for SMEs and even the entire industrial chain. Since 2015, the supply chain financial market has grown at an annual rate of 10%, reaching RMB 24.9 trillion in 2020. Supply chain finance plays an important role in supporting SMEs and serving the real economy. In fact, in September 2020, the People’s Bank of China, several ministries and commissions jointly officially promulgating the "Opinions on Regulating the Development of Supply Chain Finance to Support the Stable Cycle, Optimizing and Upgrading of the Supply Chain Industry Chain", a guideline document for promoting the standardized development of supply chain finance at the national level. This means that the development of supply chain finance has not only received strong support from national policies, but importantly, has created a favorable environment to accelerate the development of supply chain finance.

Supply chain finance is the financing behavior between enterprises that integrates the capital flow into the physical supply chain, so as to provide financing optimization for enterprises, integrate the financing process with customers, suppliers and service providers, and then enhance the value of all participants (Pfohl et al, 2009 [2]). Unlike traditional financial institutions, the main body of supply chain finance is non-financial enterprises, and the core enterprise credit is the foundation of its development. Relying on the supply chain relationship formed with core enterprises can effectively compensate for the shortage of investment funds and low credit rating of SMEs. Therefore, whether small and medium-sized enterprises can rely on the supply chain relationship formed with core enterprises, overcome the shortcomings of their poor financial and credit quality, and obtain supply chain financial support, so as to improve the efficiency of enterprise innovation has become the focus of this paper.

The rest of the structure is arranged as follows: The second part is the literature review; the third part is the theoretical mechanism analysis; the fourth part is the research design and empirical analysis; the fifth part is the heterogeneity analysis; Finally, there are research conclusions and enlightenment suggestions.

## 2 Literature review

This paper mainly examines supply chain finance and innovation efficiency. While innovation efficiency has always received a great deal of scholarly attention in the field of innovation economy research, supply chain finance research is a fairly recent field of research. As a result, only a handful of academic articles examine the direct correlation between the two. This paper sorts out relevant documents from two aspects: innovation efficiency, supply chain finance and enterprise development.

### 2.1. Efficiency of enterprise innovation

Efficiency of enterprise innovation is to obtain the largest innovation output with the smallest investment in innovation, while at the same time, improving the overall productivity of enterprises, their technical capacity, and realizing industrial upgrading according to production experience (Kontolaimou et al., 2016 [3]). The promotion and benefits of scientific and technological progress on economic and social development are pushing countries around the world to continuously increase their investment in innovation. However, an obvious fact is that the improvement of innovation efficiency is not proportional to the increase in innovation investment, and innovation is becoming more and more difficult (Nicholas et al., 2020 [4]). As the main body of innovation, enterprises are the key to improving innovation efficiency. However, at present, the innovation efficiency of many Chinese enterprises is still at a low level. For example, Wang et al. (2016) [5] found that the innovation efficiency of Chinese new energy enterprises is lower than that of other industries.

From the perspective of the process, enterprise innovation is divided into two stages: the formation and acquisition of new knowledge, and the commercialization of new knowledge. In these two stages, the differences of enterprises’ own characteristics will have different effects on their effective innovation (Martinez et al., 2019) [6].

From the perspective of internal management, the characteristics and level of internal governance of an enterprise have a great impact on innovation efficiency: the study of Shu et al. (2011) [7] shows that a moderately large-scale, diligent, responsible and shareholding a low proportion of the board of directors has a positive impact on corporate innovation efficiency for a technology-based enterprise. Generally, reducing the concentration of enterprise ownership can more effectively limit the rights of enterprise owners to a reasonable range, which is beneficial to the overall improvement of corporate innovation efficiency (Sheikh and Hofmann, 2018) [8]. Bargeron et al. (2010) [9] combined equity pledges, which is a relatively common phenomenon in Chinese listed companies, and pointed out that the hollowing out and short-sighted effects of shareholder pledges would restrict the improvement of corporate innovation efficiency.

From the perspective of the external environment of enterprises, the more consistent conclusion throughout the literature is that government subsidies (Huang et al., 2016) [10], tax incentives (Thomson, 2010) [11], industrial policies (Aghion et al., 2015) [12] and other policies have a significant role in promoting the innovation efficiency of enterprises. Considering the market environment, Hirshleifer (2013) [13] pointed out that the degree of marketization is closely and positively related to the efficiency of enterprise innovation. When the degree of market economy development is still relatively low, the distortion of factor allocation will significantly inhibit the innovation efficiency of enterprises (Boldrin and Levine, 2004) [14]. As such, in order to improve enterprise innovation efficiency (Günsel, 2015) [15], it is necessary to strengthen market design from two aspects: market thickness and market fluency.

From the perspective of market opening and development, Zhao and Fang (2019) [16] show that Chinese OFDIs have a better effect on improving innovation efficiency. Further, from the perspective of market micro-mechanisms, if enterprises want to improve innovation efficiency, they should, on the one hand, actively adopt innovative ecological strategies, rationally build an innovation ecosystem and clarify their own positioning (Liu et al., 2017) [17], and on the other hand, they should appropriately control the concentration of corporate customers to avoid the inhibition effect of innovation efficiency brought about by excessive concentration (Cao et al., 2020) [18]. For monopolistic state-owned enterprises, which have relatively low innovation efficiency, moderate mixed reform is also a possible choice to improve innovation efficiency (Gibson and Vermeulen, 2003) [19].

To sum up, the innovation efficiency of enterprises is affected by the internal and external environment of enterprises. Currently, the innovation efficiency of enterprises in China is still at a relatively low level, with great potential for improvement.

### 2.2. Supply chain finance and the development of small and medium-sized enterprises

China’s supply chain finance has developed very rapidly in recent years. This has been primarily driven and encouraged by favorable policies and the financing needs of SMEs. These trends have inspired scholarly research on the subject. The understanding of the connotation of supply chain finance can be divided into two parts: supply chain and finance. On the one hand, supply chain means that supply chain finance is carried out based on the structure of supply chain, while finance means that supply chain finance is essentially a kind of financial service developed around the credit relationship in the supply chain (Gelsomino et al., 2016) [20]. Regardless of which perspective, supply chain finance is an effective means to alleviate the liquidity pressure on enterprises (Xu et al., 2018) [21]. SMEs which have financing constraints are the main and stable demanders of supply chain finance. In addition, due to technological progress, the supply of supply chain finance is constantly evolving: starting from the initial leading model of financial institutions and core enterprises, and gradually developing to the internet platform (Song et al., 2016) [22]. Furthermore, with the development of financial technology, supply chain finance can control and manage risks by combining new technologies such as block chain to improve operational efficiency (Chod et al., 2020) [23]. To a certain extent, this change can promote the profit model evolution of supply chain financial services to better adapt to the financial service needs of small and medium-sized enterprises (Su and Zhong, 2017) [24].

From the perspective of financing, the difference between supply chain finance and traditional financing is that the credit judgement of the supply chain network replaces the credit judgement of a single enterprise, and the relationship and status of enterprises in the supply chain network system will affect financing performance (Wetzel and Hofmann, 2019) [25]. There are exchanges of information and resources between companies in the supply chain. This exchange can be divided into strong ties and weak ties according to frequency and dependence. Weak ties can reflect cross-enterprise communication to a certain extent. Indeed, it has been demonstrated that in the performance of using supply chain finance financing, weak ties companies are stronger than strong ties companies (Tomlinson, 2011) [26]. Further, from the perspective of the characteristics of SMEs embedded in the supply chain network, Autry (2011) [27] pointed out that the competitiveness of SMEs and the ability to maintain network embedding are positively related to the financing performance of supply chain finance. While from the perspective of behavioral characteristics, SMEs, which are embedded in the supply chain network, will develop two types of behaviors: utilization learning and exploratory learning when they are managing network relationships. On the one hand, supply chain relationships formed by utilization learning can significantly improve the performance of supply chain finance. While on the other hand, the supply chain financial financing performance of the supply chain relationship formed by exploratory learning is not ideal compared with the former because the company’s innovation ability will greatly compensate for the lack of financing performance which is led by exploratory relationship (Turner et al., 2013) [28]. This is especially the case when the internal innovation potential of the enterprise and the external collaborative innovation ability are coordinated development, leading to the improvement of the financing performance of the supply chain finance (Zhao et al., 2013) [29].

From the perspective of innovation, on the one hand, the innovation ability of supply chain enterprises is an important factor in the development of supply chain finance, and on the other hand, the development of supply chain finance can also affect enterprise innovation. The empirical research of Pan et al. (2021) [30] shows that supply chain finance has a significant role in promoting technological innovation of core enterprises, especially for those that are in a weak position and have severe financing constraints in the supply chain relationship. Importantly, this kind of promotion is more prominent. More broadly, the development of supply chain finance has a more obvious impact on business performance that includes corporate financing and innovation. Of course, this impact depends on the development orientation of supply chain finance: compared with the financial-oriented supply chain finance model, the supply chain orientation can effectively reduce the business risk and improve the operating efficiency of the enterprise (Moretto et al., 2019) [31].

In summary, the supply chain finance can alleviate the financial pressure of small and medium-sized enterprises and promote technological innovation of core enterprises, but it will increase corporate financial risks.

### 2.3 Literature summary

Overview of existing literature, research on innovation efficiency mainly focuses on the characteristics of enterprises and the market environment where they are located. The current development of supply chain finance has become one of the most important external environments that SMEs cannot ignore. Yet only a handful of literature focus on the impact of supply chain finance development on the innovation efficiency of SMEs. To fill this gap, this article will take supply chain finance as an entry point, focusing on the mechanism of innovation efficiency and empirical effects of its development to SMEs embedded in the supply chain network.

Compared with the existing research, the marginal contributions of this paper are: (1) From the perspective of theoretical mechanism, previous research often focuses on the positive impact of supply chain finance on enterprise innovation efficiency, and it is relatively one-sided. This paper discusses the impact of supply chain finance on the innovation efficiency of enterprises from both positive and negative sides (see theoretical mechanism for details), and enriches the theoretical research related to supply chain finance to a certain extent. (2) From the perspective of research objects, previous research industries lacked pertinence and often concentrated on core companies in the supply chain. This paper focuses on the audience of small and medium-sized manufacturing enterprises, and conducts detailed research based on industrial attributes and property rights characteristics. (3) In terms of policy, this paper finds that supply chain finance has obvious differences in the innovation efficiency of manufacturing SMEs with different industrial attributes and property rights characteristics, which is conducive to the government to better guide and timely adjust the development of supply chain financial services, and promote my country’s manufacturing industry. The innovation level of small and medium-sized enterprises has been improved.

## 3 Theoretical mechanism

The research in this paper is based on the hypothesis that supply chain finance has a positive and negative impact on the innovation efficiency of SMEs. The theoretical mechanism analysis clarifies the specific impact methods, which provides a theoretical basis for the empirical analysis results below.

### 3.1. Supply chain finance promotes open innovation efficiency of small and medium-sized enterprises

It should be noted from the outset that the development of supply chain finance relies on a healthy and stable supply chain. SMEs are embedded in the supply chain network to exchange resources and information based on the supply chain relationship, which provides them with great opportunities to open innovation (Fu et al., 2021) [32]. This is done in several ways. First, the embedded supply chain network increases the supply of external innovation factors for SMEs. Although knowledge is the basic element of innovation, SMEs are often slow in accumulating it and possess relatively fewer channels for obtaining innovative information from outside. With the development of science and technology, the frequency of knowledge updating is also accelerating. As such, obtaining innovation knowledge more quickly and accurately has become a key issue in improving the innovation efficiency of SMEs (Zhang et al., 2018) [33].

After an enterprise joins the supply chain, relying on the information exchange generated by mutual trade, it is possible to observe the update of the innovation knowledge of upstream and downstream enterprises in the supply chain system, which provides an opportunity for SMEs to grasp and track the latest innovation knowledge. At the same time, the financing services based on the supply chain relationship provide strong financial support for SMEs to acquire new knowledge, thus alleviating the difficulty of obtaining innovative knowledge due to financing constraints.

Secondly, being embedded in the supply chain network helps SMEs optimize their innovation process through behaviors such as using, learning and exploring network relationships (Chakuu et al., 2019) [34]. In doing so, they can observe the innovation process of other enterprises on the network. Importantly, when SMEs rely on the supply chain to be bundled with the value of other enterprises, when other enterprises carry out innovative activities, they may carry out joint activities with SMEs, by referring to enterprises with high efficiency of innovation activities in the supply chain, SMEs can learn from the innovation process and factor investment of other enterprises, reduce the invalid links in innovation activities, and then improve their innovation efficiency (Li, 2020 [35]; Li and Shi, 2020 [36]).

Third, using supply chain financial services can help SMEs to efficiently transform innovative elements into commercial value (Lu et al., 2020) [37]. Supply chain financial services are accommodation of funds based on the supply chain relationship. Usually manifested as a shorter time and smaller scale. The financial services are provided through multiple batches of rapid cycles, and sometimes the cost is slightly higher than that of traditional financing. Obviously, the basis for continuously providing good supply chain financial services is still the effective operation of enterprises. In the current knowledge economy, SMEs that want to obtain more benefits can rely on innovation activities, i.e., they can effectively transform innovative elements such as knowledge into commercial values. After being embedded in the supply chain network, SMEs can, on the one hand, optimize the production process, improve product quality, reduce production costs and timely implement effective process innovation according to the requirements of the suppliers, and on the other hand, they can use the feedback from consumers, which are transmitted directly by supply chain network, to evaluate the commercial value realization possibility and size of innovation. Driven by the goal of promoting the effective circulation of financial services in the supply chain, the efficiency of innovation behavior is ultimately improved by realizing the commercial value brought by innovation.

### 3.2. The core enterprise innovation spillover of supply chain finance helps small and medium-sized enterprises to improve their innovation efficiency

Core enterprises are the basic nodes of supply chain financial services, and their innovative behavior has a significant spillover effect on the improvement of innovation efficiency SMEs that are supported by supply chain financial services. First, the innovation activities of core enterprises provide upstream and downstream SMEs with incentive to improve their innovation efficiency (Liu et al., 2013) [38]. Core companies in the supply chain financial system are generally companies which own a strong position in the industry. Their knowledge, technology, talent, product and market advantages, often make their innovation activities more likely to be efficient and have a higher probability of success. This will have a strong leading and demonstration effect on the innovation of upstream and downstream SMEs in the supply chain network: they can organize their own research input by closely following the innovation direction of core enterprises, effectively avoiding innovation directional errors, and reducing the cost of trial and error in innovation, and ultimately improve the input efficiency of innovation elements.

Second, the innovation activities of core enterprises increase the flexibility of innovation funds for upstream and downstream SMEs (Chen et al., 2015) [39]. The sufficient capital of core enterprises is the base of maintaining the healthy circulation of the supply chain financial system. Core companies are usually innovation leaders in the industry, and most of them can realize innovative value through effective innovation. The financial success achieved by innovation enables core enterprises to provide more reliable liquidity guarantees when solving the financing constraints of SMEs in the supply chain network. From the perspective of collaborative innovation, core enterprises lead the collaborative innovation of SMEs in the supply chain network, which can jointly resist the uncertainty faced by innovation and reduce innovation costs, thus improving the financial performance of the entire supply chain instead of a single core enterprise and enhance the credit of SMEs embedded in the supply chain network effectively. Further SMEs embedded in the supply chain network and participating in collaborative innovation can arrange the funds for innovation more reasonably capital that are based on innovation and greatly promote the success rate of innovation by sharing the innovation resources and information of core enterprises.

Third, the innovation of core enterprises’ supply chain financial services has improved the innovation efficiency of upstream and downstream SMEs (Xia et al., 2020) [40]. The supply chain financial services derived from the trade credits provided by core enterprises are constantly innovating and developing under the advancement of science and technology and are also continuously improving the innovation efficiency of small and medium-sized enterprises embedded in the supply chain network. In the process of innovation and development, the core enterprises of supply chain financial services have gradually transitioned to platform-based institutions. This change has deepened the innovative synergy association between the core enterprises in the supply chain network and upstream and downstream SMEs and enriched the latter’s channels to obtain innovative resources. In other words, it shortens the time needed to realize the value of innovation of SMEs and promotes their improvement of innovation efficiency. Innovative development is also expanding the supply chain financial tool shed, from early inventory pledges, advance receipts and payable financing to factoring and reverse factoring, etc. The acting objects are also deepening from trade funds to working capital. This change optimizes the risk management methods of supplying financial services, expands the freedom of SMEs to obtain innovative resources, and enables them to use supply chain financial services from the trade field to the production and operation field, then improve the role of supply chain financial services in promoting the breadth and depth of innovation activities.

### 3.3. Competition and cooperation relationship in the supply chain financial system promote the innovation efficiency of small and medium-sized enterprises

The various entities in the supply chain communicate with each other through logistics, information and trade flow, and finally form a relatively stable supply chain relationship, which includes mainly cooperative and competitive relationships (Reza-Gharehbagh et al., 2021) [41]. In the non-central supply chain, the participating entities mainly achieve cooperation through mechanisms such as supply chain contracts, information exchanges, and joint decision-making. Conversely, in the central supply chain, the participating entities mainly cooperate through mechanisms such as price, repurchase, benefit sharing contracts, service level contracts, discount policies, and credit cycle conditions. Further, in the non-central supply chain cooperation relationship, it is better for supply chain members to maximize the benefits of the supply chain rather than the individual enterprise. Therefore, the main ways for members to cooperate is through developing products jointly and exchanging information with one another. In this way, SMEs embedded in the supply chain network can achieve the exchange of innovation resources, the mutual learning of innovation processes, the sharing of innovation results, and the improvement of their own innovation efficiency through the collaborative innovation of the entire supply chain. Under the conditions where the entire supply chain faces financing constraints, chiefly as a result of pressure of the interests of external capital providers, the efficiency of this integration of resources across the chain for innovation will be higher after realizing external financing through the supply chain relationship. In the central supply chain mode, the core enterprises often manage the supply chain resources centrally. In order to achieve more favorable resource allocation, core enterprises often choose SMEs with high innovation efficiency to cooperate more closely, which encourages them to strive to improve innovation efficiency (Bessonova and Gonchar, 2019) [42].

The supply chain financial system where funds drive the circular development of the supply chain is usually embedded in the business ecosystem. Catalyzed by technological development, the supply chain financial ecosystem also has dynamical evolution (Lam et al., 2019) [43]. When a major technological update occurs in the industry, it is often accompanied by the adjustment and reconstruction of the supply chain relationship (Minniti, 2010) [44]. In order to maintain and enhance the company’s position in the supply chain financial system when potential changes occur, SMEs embedded in the supply chain financial system also must compete. This is first manifested in the competition for the position of innovation leader (Bals., 2019) [45]. The benefits of managing capital flows in supply chain finance depend on the competitive position based on innovation capabilities. To maximize the benefits of supply chain financial services, SMEs must innovate their catch-up strategies and maintain relative competition advantages with other SMEs in the supply chain network. This pushes SMEs to continuously strengthen their innovation ability and improve innovation efficiency (Theeke, 2016) [46]. When rebuilding the supply chain financial system, this kind of competition is reflected in the competition between existing companies in the supply chain and potential competitors. The reconstruction of the supply chain financial system means that potential entrants may replace the enterprises in supply chain (Tang, 2006) [47]. To actively maintain the existing position of the supply chain financial system and avoid being eliminated by potential entrants, SMEs that have been embedded in the supply chain financial system must improve innovation efficiency actively and maintain a competitive advantage over external substitutes (Cui et al., 2020) [48].

### 3.4. The spread of internal risk factors in supply chain finance inhibits the improvement of small and medium-sized enterprises’ innovation efficiency

Innovation activities often require a large amount of funds as support. Large-scale innovation activities may cause two main problems: funds cannot flow, and debts cannot be paid in time, which is caused by the insufficient debt repayment ability of innovative enterprises. This in turn leads to financial risks such as supply chain funding gaps and failure to connect supply chain funds (Kouvelis and Zhao, 2016) [49].

At the same time, due to the expansion of the supply chain, the previous core enterprises may not have the ability to support all the funds in the supply chain. In the case of information asymmetry and in order to avoid the emergence of problems such as adverse selection, some core enterprises may use their dominant position in the supply chain to increase the accounts payable and advance receipts to upstream and downstream enterprises. This kind of unequal status in the supply chain and concerns about the financial strength can easily trigger credit risks in the supply chain, making it more difficult for innovative enterprises to raise funds (Yang and Birge, 2018) [50].

With the development of the supply chain industry, more and more enterprises or financial institutions participate in the supply chain. With the increase of supply chain participants and the frequent communication between supply chain members, it is gradually combined into a community of interests, and in this process, the mutual exchanges between enterprises gradually form a supply chain network structure, which increases the complexity of the supply chain. As a result of this kind of network structure, supply chain financial risks are strengthened and spread rapidly, the financial risk or credit risk of supply chain enterprises will be transmitted to other related enterprises through the supply chain. This phenomenon is more obvious in core enterprises. When enterprises carry out innovative activities, this kind of behaviors will aggravate the financing constraints faced by innovative enterprises, thus inhibiting innovation efficiency.

### 3.5. The imperfect external environment of the supply chain leads to higher financing costs for innovation enterprises and inhibits innovation efficiency

First, supply chain finance involves a large number of enterprises and covers a wide range of industries, making the overall supply chain relatively riskier and more difficult to control risk (Heckmann et al., 2015) [51]. From a legal perspective, it is manifested as imperfect laws and regulations. In the past, laws and regulations mainly regulated single enterprise entities and transaction behaviors, but they lacked corresponding standards and constraints on supply chain finance. For example, in the very important mortgage part of supply chain financing, there are still some problems, such as narrow mortgage scope, repeated mortgage and unclear right to dispose of collateral. The appearance of legal risks will hinder the construction of a supply chain financial credit risk evaluation system to a certain extent.

Second, from an institutional perspective, although supply chain finance has been actively promoted in recent years, there are still relatively few specific policy measures, and the implementation of policy measures has a certain time lag—making it difficult for new policies to adapt to the ever-changing supply chain financial situation (Kiefer et al., 2019) [52]. And as an important supervisory authority, it is often responsible for regulating and reviewing supply chain financial behavior. At present, the government appears to be absent in the supervision of local supply chain finance and the channels for obtaining information are limited. As a result, it is impossible to track the dynamics of enterprises. Furthermore, loose management may cause supply chain enterprises to maximize their benefits by increasing accounts receivable and strengthening the requirements for pledges.

As a result of the emergence of external factors such as imperfect laws and lack of institutional supervision, enterprises often require certain physical inventories, warehouse receipts, receivables, prepayments, etc. as pledges or mortgages for the consideration of sharing supply chain financial risks. In practice, supply chain finance mostly takes bulk commodities as pledge assets (Chiu and Choi, 2013 [53]; Tang et al., 2018 [54]). However, innovative products are difficult to be recognized by other enterprises or financial institutions in the supply chain to be a suitable pledged commodity due to their own characteristics such as large price fluctuations, long cycles, strong seasonality, and high risks, coupled with the information asymmetry between enterprises. Even if innovative products are accepted as collateral, the large fluctuations in the prices of innovative products may also trigger the overall systemic risk of the supply chain. Due to the limitations of innovative products as collateral or pledges, in order to meet financing needs, innovative enterprises often need other assets as supplements, which weakens the impact of supply chain finance on innovation efficiency.

### 3.6. Adverse selection in supply chain finance leads to resource misallocation and inhibits innovation efficiency

**Fig 1:**
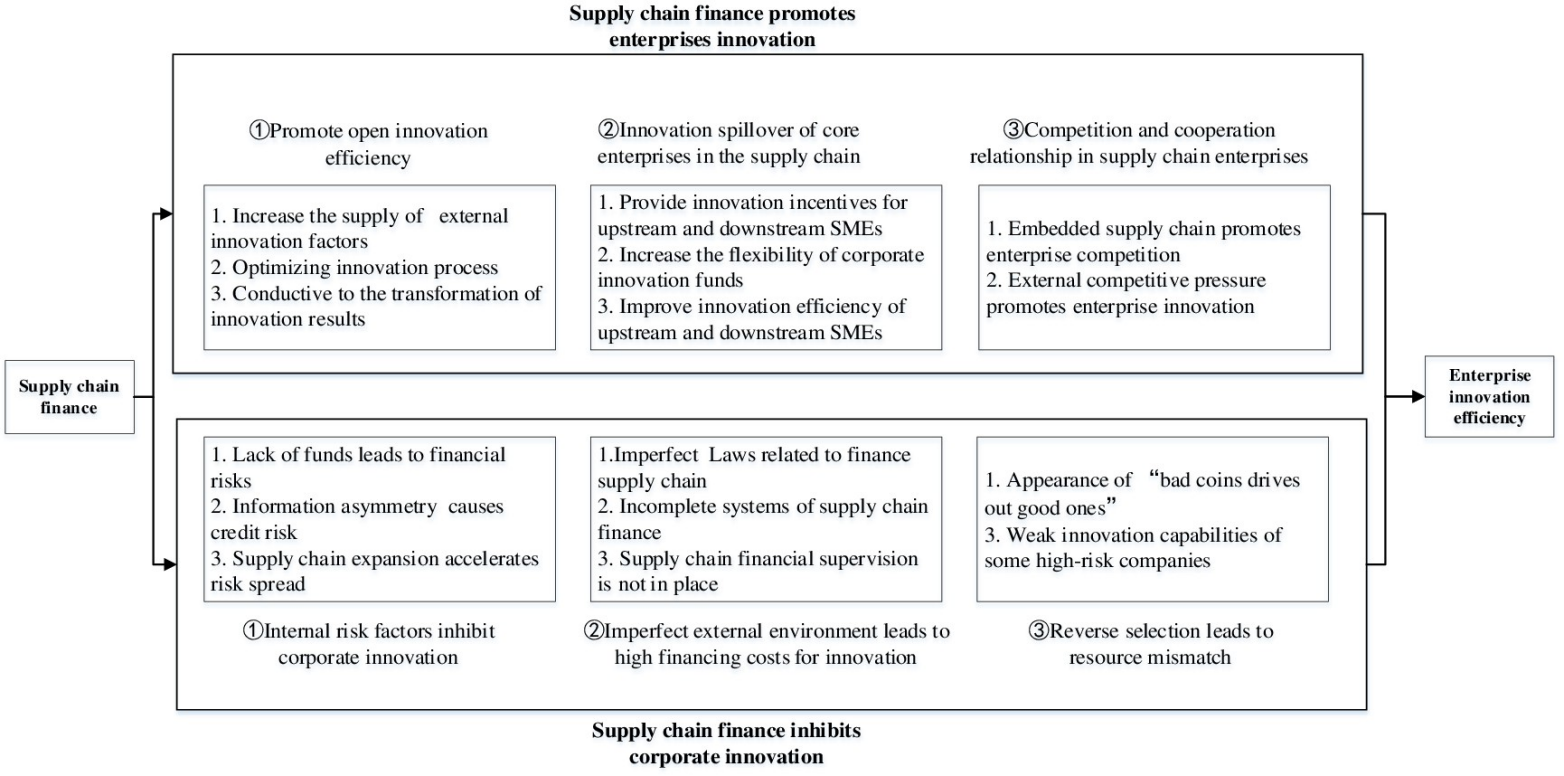
Theoretical mechanism of supply chain finance affecting enterprise innovation efficiency.

Through field research and interviews, this paper found that the lack of a good information exchange platform, supply chain enterprises tend to have a certain degree of information asymmetry between each other, which is more obvious in terms of corporate financial status. This situation often leads to adverse selection in supply chain finance. Moreover, enterprises with poor financial state tend to be the party that most needs supply chain finance. Such enterprises have the motive to deliberately cover up their poor financial situation in order to obtain the support of supply chain finance. However, the amount of funds in the supply chain is limited, and enterprises that are truly capable of innovating may not be able to obtain financial support, resulting in the phenomenon of "bad money driving out good money". Moreover, high-risk enterprises have a hard time to effectively improve innovation efficiency because of meager finances. If they encounter risk, it will spread to the entire supply chain network through supply chain finance, thereby reducing the innovation efficiency of the overall supply chain network. Finally, in order to facilitate observation, we build the structure of this paper (Fig 1).

## 4 Sample and variables

### 4.1. Research design

Based on the analysis of the theoretical mechanism mentioned above, this article intends to conduct an empirical analysis with China’s small and medium-sized manufacturing industry as the observation object. This is motivated by two reasons: first, the high-quality development of the Chinese manufacturing industry is the muain pillar which will support the high-quality development of China’s economy in the future, and international trade frictions and technological sanctions have clearly proved this point in recent years. Second, not only is the supply chain system of China’s manufacturing industry not mature, but also, the application of supply chain finance in the manufacturing sector remains insufficient. Therefore, it is necessary to conduct an in-depth investigation of the mechanism of supply chain finance and manufacturing development. The design is as follow:

#### Sample selection

SMEs are in a weak position in the supply chain, but they have a strong need for support from supply chain finance. To adapt to market changes, SMEs have higher enthusiasm in innovation to adapt to market changes Given the availability of data, this article selects listed enterprises in the manufacturing industry (Class C listed enterprises on China Growth Enterprise Market) in the growth enterprises board as the sample and removes some samples of companies with serious missing data, then finally it selects 267 listed companies in the China Growth Enterprise Market as the sample for investigation. From an industrial perspective, it is divided into high-tech industry enterprises (152) and traditional manufacturing enterprises (115). High tech industry enterprises often have higher innovation capabilities than traditional industry enterprises. From the perspective of property rights, there are 14 state-owned enterprises and 253 private enterprises. State owned enterprises often have strong risk resistance capabilities, while private enterprises are relatively weak. Due to the short time of the establishment of the growth enterprises board, the time span selected in this article is 2015–2019, with a total of 1335 valid sample data.

Among them, the high-tech industries involved in the sample enterprises are pharmaceutical manufacturing, computer, communications and other electronic equipment manufacturing, railway, shipbuilding, aerospace and other transportation equipment manufacturing, instrumentation manufacturing, professional technical service industry, and special equipment manufacturing.

#### Measurement of innovation efficiency of SMEs

In this paper, the ratio of the number of R&D personnel to the total number of enterprises is taken as the human capital investment of enterprise innovation, and the ratio of R&D investment to operating income is taken as the capital investment of enterprise innovation, then select the amount of enterprise patent authorization in the current year as the final output of enterprise innovation, measure the comprehensive innovation efficiency (CE) of manufacturing SMEs through the DEA-SBM method, and decompose it into organizational innovation efficiency (OE) and technological innovation efficiency (TE). All data can be found in the Cathay Pacific Database.

#### Supply chain finance measurement

The development level of supply chain finance (SCF) is represented by the ratio of the sum of short-term loans and bills payable to the total assets of the enterprise. The amount of short-term loans represents the loans that enterprises borrow from financial institutions for a period of one year in order to alleviate their short-term capital needs; while bills payable are commercial acceptances and banker’s acceptances that should be paid by small and medium-sized manufacturing enterprises. Divide the sum of the two by the total assets. Prevent the measurement bias of empirical results caused by differences in company size.

### 4.2. Basic model construction

Considering the accuracy of efficiency measurement, this paper uses the DEA-SBM method to measure the comprehensive innovation efficiency (CE) of manufacturing SMEs, and decomposes it into organizational innovation efficiency (OE) and technological innovation efficiency (TE). All three efficiencies are used as explained variables in this paper for empirical analysis. This article draws on the measurement method of Yao (2017) [55], taking the ratio of the sum of short-term borrowings and bills payable to the total assets of manufacturing enterprises as a measure of supply chain finance (SCF) and SCF is used as an explanatory variable for empirical analysis in this paper. Considering the influence brought by the company’s own factors, the common practice in the reference literature is to choose the natural logarithm of the company’s total assets (TA), return on enterprise total assets (RNA), the number of years from the date of establishment of the enterprise to the observation period (AGE), earnings per share (EPS), equity multiplier (EM) is used as a control variable, and the interpolation method is used in this paper to deal with the vacancies in the indicators. The description of the indicators selected for the variable selection is summarized in Table 1 below:

**Table 1:** Variable definition table.

Variable type	Variable name	Variable definitions	Data Sources
Explained variable	CE	Comprehensive innovation efficiency measured based on DEA-SBM method	Cathay Pacific Database, CCER database
	OE	Organizational innovation efficiency decomposed based on the total efficiency measured by the DEA-SBM method	
	TE	Technological innovation efficiency decomposed based on the total efficiency measured by the DEA-SBM method	
Explanatory variables	SCF	The ratio of the sum of short-term loans and bills payable to the total assets of the enterprise.	Cathay Pacific Database
Control variable	TA	The natural logarithm of the company’s total assets	
	RNA	Return on enterprise total assets	
	AGE	The number of years from the date of establishment of the enterprise to the observation period	
	EPS	Earnings per share	
	EM	Equity Multiplier	

Model construction. Based on the above theoretical analysis, variable design and selection process, this article constructs the following equation to empirically analyze the impact of supply chain financial services on the innovation efficiency of small and medium-sized manufacturing enterprises:

$$CE_{it}=\alpha_{0}+\alpha_{1}SCF_{it}+\alpha_{k}\sum control_{it}+\nu_{it}$$ (1)

$$OE_{it}=\delta_{0}+\delta_{1}SCF_{it}+\delta_{k}\sum control_{it}+\eta_{it}$$ (2)

$$TE_{it}=\varphi_{0}+\varphi_{1}SCF_{it}+\varphi_{k}\sum control_{it}+\tau_{it}$$ (3)

Among them, *CE*_*it*_, *OE*_*it*_ and *TE*_*it*_ respectively represent the comprehensive innovation efficiency, scale innovation efficiency, and technological innovation efficiency. *α*_0_, *δ*_0_ and *φ*_0_ are constants. *SCF*_*it*_ representing the supply chain finance level of enterprise, while *α*_1_, *δ*_1_, *φ*_1_ respectively represent the impact of supply chain finance on comprehensive innovation efficiency, scale innovation efficiency, and technological innovation efficiency. *control*_*it*_ represents various control variables in the model, including the natural logarithm of the company’s total assets(TA), return on total assets (RNA), the number of years from the date of establishment of the enterprise to the observation period(AGE), earnings per share (EPS), and equity multiplier (EM). *α*_*k*,_ *δ*_*k*_ and *φ*_*k*_ representing the impact of control variables on comprehensive innovation efficiency, scale innovation efficiency technological innovation efficiency and technological innovation efficiency. *ν*_*it*_, *η*_*it*_ and *τ*_*it*_ respectively represent the random errors of the model.

## 5 Empirical analysis

### 5.1. Descriptive statistics of innovation efficiency of small and medium-sized manufacturing industries

The results of innovation effectiveness of small and medium-sized manufacturing enterprises can be obtained from the calculation results of MYDEA software. Tables 2–4 respectively record the statistical characteristics of the innovation efficiency of the three types of samples for the full sample, the sub-sample based on industry attributes, and the sub-sample based on property rights attributes. As Table 2 shows, the average value of the innovation efficiency of the total sample showed a downward trend from 2015 to 2017, with a sudden large, yet temporary, increase in 2018, before falling below the 2017 level by 2019. In addition to the sample of state-owned enterprises, this trend is also reflected in other samples.

**Table 2:** Statistics of innovation efficiency for the full sample.

Full sample statistics							
	years	Mean	Max	Minimum	Median	Standard deviation	Dispersion coefficient
**Comprehensive innovation efficiency**	2015	0.111	1	0.003	0.064	0.147	1.326
	2016	0.107	1	0.004	0.064	0.149	1.392
	2017	0.106	1	0.002	0.059	0.146	1.378
	2018	0.131	1	0	0.064	0.183	1.401
	2019	0.104	1	0	0.052	0.165	1.579
**Organizational innovation efficiency**	2015	0.441	1	0.038	0.41	0.265	0.601
	2016	0.290	1	0.022	0.241	0.216	0.745
	2017	0.289	1	0.018	0.22	0.239	0.826
	2018	0.350	1	0	0.301	0.264	0.756
	2019	0.258	1	0	0.192	0.254	0.984
**Technological innovation efficiency**	2015	0.213	1	0.014	0.173	0.167	0.784
	2016	0.319	1	0.042	0.283	0.173	0.541
	2017	0.328	1	0.057	0.291	0.175	0.533
	2018	0.301	1	0.055	0.253	0.195	0.648
	2019	0.340	1	0.08	0.316	0.179	0.527

**Table 3:** Sub-sample innovation efficiency statistics based on industry attributes.

		Traditional industry sample			High-tech industry sample		
	years	Mean	Standard deviation	Dispersion coefficient	Mean	Standard deviation	Dispersion coefficient
**Comprehensive innovation efficiency**	2015	0.119	0.158	1.321	0.104	0.138	1.327
	2016	0.112	0.151	1.343	0.104	0.149	1.436
	2017	0.109	0.151	1.383	0.104	0.143	1.377
	2018	0.138	0.189	1.376	0.125	0.178	1.425
	2019	0.11	0.17	1.543	0.1	0.161	1.612
**Organizational innovation efficiency**	2015	0.402	0.257	0.639	0.47	0.268	0.57
	2016	0.271	0.21	0.775	0.305	0.22	0.722
	2017	0.262	0.225	0.861	0.309	0.247	0.799
	2018	0.332	0.252	0.762	0.363	0.273	0.751
	2019	0.238	0.244	1.025	0.274	0.262	0.955
**Technological innovation efficiency**	2015	0.243	0.18	0.742	0.19	0.152	0.802
	2016	0.358	0.18	0.504	0.289	0.161	0.556
	2017	0.367	0.186	0.508	0.299	0.16	0.535
	2018	0.337	0.207	0.615	0.274	0.181	0.662
	2019	0.385	0.192	0.497	0.305	0.161	0.527

**Table 4:** The new efficiency statistics table based on the attributes of property rights.

		State-owned enterprise sample			Private enterprise sample		
	years	Mean	Standard deviation	Dispersion coefficient	Mean	Standard deviation	Dispersion coefficient
**Comprehensive innovation efficiency**	2015	0.105	1.269	0.112	0.149	1.325	0.083
	2016	0.092	1.219	0.109	0.152	1.392	0.075
	2017	0.045	0.781	0.109	0.149	1.373	0.058
	2018	0.176	1.327	0.130	0.184	1.408	0.132
	2019	0.126	1.267	0.105	0.167	1.595	0.100
**Organizational innovation efficiency**	2015	0.256	0.575	0.440	0.266	0.604	0.446
	2016	0.218	0.806	0.291	0.216	0.743	0.271
	2017	0.196	0.770	0.291	0.241	0.829	0.254
	2018	0.266	0.667	0.347	0.265	0.762	0.400
	2019	0.257	0.814	0.255	0.254	0.996	0.315
**Technological innovation efficiency**	2015	0.105	0.668	0.216	0.169	0.783	0.158
	2016	0.127	0.532	0.323	0.174	0.538	0.239
	2017	0.104	0.433	0.333	0.177	0.531	0.239
	2018	0.184	0.718	0.304	0.196	0.645	0.256
	2019	0.131	0.448	0.342	0.181	0.529	0.292

However, on average, the comprehensive innovation efficiency of traditional industries is higher than that of high-tech industries. This may be because high-tech industries, which work as technology-intensive industries, have more difficulties to achieve large-scale innovations and breakthroughs. The innovation efficiency of private enterprises is also significantly higher than that of state-owned enterprises. This may be because private enterprises are facing fierce market competition and need to continuously improve their innovation level to maintain competitiveness. However state-owned enterprises may be supported by the government and tend to be in a favorable position in market competition, resulting in insufficient motivation for their own innovation.

From the point of view of standard deviation and dispersion coefficient, except for the sample of state-owned enterprises, the rest are showing a gradual upward trend, which shows that the difference in innovation efficiency between enterprises is gradually widening. In the sample of state-owned enterprises, except for the decline in 2017, the dispersion coefficient remained basically stable for the rest of the time and was significantly smaller than other samples. This shows that the difference in comprehensive innovation efficiency among state-owned enterprises is relatively stable.

As illustrated in Table 3, the organizational innovation efficiency shows a trend of declining year by year from the point of average value, and the trend of the sample of traditional industries is more obvious. Compared with other samples, the organizational innovation efficiency of traditional industries is relatively high. In 2018, the organizational innovation efficiency of traditional industries reached the highest level in the sample during the same period, both in terms of growth rate and numerical value.

Dispersion coefficient reflects the differences in innovation efficiency between enterprises. Horizontally, we can see that the dispersion coefficient between comprehensive innovation efficiency and technological innovation efficiency of high-tech industries is greater than that of traditional industries, while organizational innovation efficiency is lower than that of traditional industries, which also reflects the stronger innovation vitality of high-tech industries. Vertically, the overall innovation efficiency shows a slow upward trend except in 2018. One possible explanation is that the differences in innovation efficiency among enterprises are decreasing due to trade frictions between China and the United States.

Furthermore, it can be seen from Table 4 that technological innovation efficiency is different from comprehensive innovation efficiency and organizational innovation efficiency. The average value of each sample shows an overall upward trend. The upward trend of private enterprises and traditional industries is more obvious than that of state-owned enterprises and high-tech industries. It shows that the increase in innovation efficiency of the two may be caused by the enterprise’s own factors during the observation period.

From the point of view of the dispersion coefficient, the dispersion coefficient between each sample gradually decreases. Although the standard deviation has increased during the observation period, the decrease in the dispersion coefficient seems to better reflect the gradual decrease in the difference in the technological innovation efficiency of the enterprises among the samples than the decrease in the dispersion coefficient.

### 5.2. Empirical analysis of supply chain financial services and enterprise innovation efficiency

Based on the models (1)-(3), this paper conducts an empirical analysis on the innovation efficiency of supply chain financial services and manufacturing SMEs, and firstly conducts a full sample analysis in the analysis process. The sample data selected in this paper belong to the balanced panel data. Therefore, this paper conducts time-individual double-fixed regression analysis based on the balanced panel. At the same time, in order to prevent the impact of outliers in the sample on the empirical results, this paper adopts 1% of the main variables before and after shrinking. Based on the results of the Hausman test(21.65,19.78,88.95), fixed effects should be used in this model. The empirical results are summarized in Table 5 below.

**Table 5:** Table of full sample estimation results.

Model	(1)		(2)		(3)	
Explained Variables	CE		TE		OE	
SCF	0.101**	0.119**	0.125***	0.093*	0.086	0.154*
	(2.51)	(2.24)	(2.76)	(1.87)	(1.39)	(1.93)
TA		0.043***		0.032*		0.053**
		(2.63)		(1.85)		(2.41)
RNA		0.128***		0.237***		0.089
		(3.46)		(5.99)		(1.37)
AGE		-0.009***		0.026***		-0.054***
		(-3.09)		(7.51)		(-9.24)
EPS		0.220**		0.218**		0.122
		(2.50)		(2.43)		(0.96)
EM		0.006		0.025		-0.022
		(0.28)		(1.30)		(-1.01)
Constant	0.097***	-0.833**	0.197***	-0.634*	0.429***	-0.522
	(18.73)	(-2.48)	(30.16)	(-1.81)	(35.51)	(-1.15)
Individual effect	Control					
Time effect	Control					
Number of samples	1,335					

Note:

*, **, *** correspond to the significance level of 10%, 5%, and 1% respectively

The results of models (1)-(3) in Table 5 show that when no control variables are introduced, supply chain financial services have a significant positive impact on the comprehensive innovation efficiency and technological innovation efficiency of enterprises. After the introduction of control variables, supply chain finance not only has a significant impact on comprehensive innovation efficiency and technological innovation efficiency, but also has a significant positive effect on the organizational innovation efficiency of enterprises. According to the above theoretical mechanism, such results may be explained as: when an enterprise participates in supply chain finance, it obtains the capital and technical support required for innovation to a certain extent, thereby improving the comprehensive innovation efficiency of the enterprise. At the same time, in the process of communication between the two sides of the supply chain, enterprises can improve the innovation process and learn from the innovation mechanism by observing other enterprises, so as to improve the organizational ability of enterprises. As a result, the influence coefficient of Supply Chain Finance on organizational innovation efficiency is greater than that of technological innovation efficiency.

From the perspective of control variables, enterprise asset scale and enterprise profitability also have a significant positive effect on the three types of innovation efficiency. According to the theoretical mechanism, the possible explanation is: the larger the scale of the enterprise and the stronger the profitability, it will first show more flexible capital allocation. Secondly, with the improvement of the position of the enterprise in the supply chain, there are more opportunities for joint innovation through mutual communication with other enterprises in the supply chain, which makes the improvement of innovation efficiency more significant. It is worth noting that the establishment years of enterprises have a significant negative impact on the comprehensive innovation efficiency and organizational innovation efficiency of enterprises, but a significant positive impact on the efficiency of technological innovation. This paper holds that after a long period of development, the organization of some enterprises usually tends to be stable, and they often encounter great resistance when changing the organizational structure. However, because enterprises have been rooted in a certain market for many years, they may have more advantages in developing new technologies than new enterprises.

### 5.3. Robustness test

#### Test of supply chain finance change variables

This paper uses the logarithm of the net accounts receivable to represent the level of enterprise supply chain finance and conducts the experiment again. From the results of models (1)-(3) in Table 6, it can be seen that when no control variables are introduced, the comprehensive innovation of supply chain finance to enterprises The effects of efficiency, technological innovation efficiency and organizational innovation efficiency are significantly positive. After the introduction of control variables, in addition to organizational innovation efficiency, the impact of supply chain finance on comprehensive innovation efficiency and technological innovation efficiency is also significantly positive, which is basically consistent with the empirical results in Table 5.

**Table 6:** Robustness test result table.

Inspection method	Change variables						Use random effects model					
Model	(1)		(2)		(3)		(4)		(5)		(6)	
Explained Variables	CE		TE		OE		CE		TE		OE	
SCF	0.033***	0.018*	0.042***	0.028***	0.030*	0.014	0.129***	0.112**	0.231***	0.142***	0.051	0.097
	(2.88)	(1.87)	(3.42)	(2.83)	(1.78)	(0.84)	(3.53)	(2.24)	(4.95)	(2.87)	(0.78)	(1.26)
TA		0.026**		0.011		0.037*		0.055***		0.058***		0.063***
		(1.99)		(0.74)		(1.88)		(4.34)		(4.43)		(4.16)
RNA		0.132***		0.224***		0.106		0.134***		0.288***		0.060
		(3.49)		(5.67)		(1.54)		(3.57)		(7.45)		(0.97)
AGE		-0.009***		0.026***		-0.054***		-0.008***		0.011***		-0.033***
		(-3.01)		(7.59)		(-9.11)		(-3.24)		(3.70)		(-8.06)
EPS		0.210**		0.210**		0.110		0.168***		0.133**		0.143
		(2.42)		(2.36)		(0.87)		(2.85)		(2.04)		(1.55)
EM		0.016		0.030*		-0.007		0.014		0.032*		-0.011
		(0.89)		(1.67)		(-0.37)		(0.72)		(1.79)		(-0.48)
Constant	-0.523**	-0.826**	-0.594**	-0.732*	-0.129	-0.444	0.094***	-1.095***	0.268***	-1.102***	0.318***	-0.870***
	(-2.35)	(-2.29)	(-2.50)	(-1.95)	(-0.40)	(-0.94)	(11.14)	(-4.30)	(25.42)	(-4.20)	(21.59)	(-2.86)
Number of samples	1335											
Individual effect	Control						No					
Time effect	Control						No					

Note:

*, **, *** correspond to the significance level of 10%, 5%, and 1% respectively

However, on average, the comprehensive innovation efficiency of traditional industries is higher than that of high-tech industries. This may be because high-tech industries, which work as technology-intensive industries, have more difficulties to achieve large-scale innovations and breakthroughs. The innovation efficiency of private enterprises is also significantly higher than that of state-owned enterprises. This may be because private enterprises are facing fierce market competition and need to continuously improve their innovation level to maintain competitiveness. However state-owned enterprises may be supported by the government and tend to be in a favorable position in market competition, resulting in insufficient motivation for their own innovation.

From the point of view of standard deviation and dispersion coefficient, except for the sample of state-owned enterprises, the rest are showing a gradual upward trend, which shows that the difference in innovation efficiency between enterprises is gradually widening. In the sample of state-owned enterprises, except for the decline in 2017, the dispersion coefficient remained basically stable for the rest of the time and was significantly smaller than other samples. This shows that the difference in comprehensive innovation efficiency among state-owned enterprises is relatively stable.

As illustrated in Table 3, the organizational innovation efficiency shows a trend of declining year by year from the point of average value, and the trend of the sample of traditional industries is more obvious. Compared with other samples, the organizational innovation efficiency of traditional industries is relatively high. In 2018, the organizational innovation efficiency of traditional industries reached the highest level in the sample during the same period, both in terms of growth rate and numerical value.

Dispersion coefficient reflects the differences in innovation efficiency between enterprises. Horizontally, we can see that the dispersion coefficient between comprehensive innovation efficiency and technological innovation efficiency of high-tech industries is greater than that of traditional industries, while organizational innovation efficiency is lower than that of traditional industries, which also reflects the stronger innovation vitality of high-tech industries. Vertically, the overall innovation efficiency shows a slow upward trend except in 2018. One possible explanation is that the differences in innovation efficiency among enterprises are decreasing due to trade frictions between China and the United States.

### 5.4. Heterogeneity test

In order to better play the role of supply chain finance in improving the innovation efficiency of manufacturing SMEs, it is necessary to distinguish enterprises with different characteristics and analyze their regression characteristics. Therefore, this paper distinguishes the sample companies from the attributes of the industry and the attributes of property rights. From the perspective of industry, this paper further divides the full sample into traditional manufacturing enterprises and high-tech manufacturing enterprises for sub-sample regression. On the one hand, when enterprises engage in high-tech manufacturing, innovation breakthroughs may be more resistant than traditional manufacturing enterprises. It is necessary to consider whether high-tech manufacturing enterprises can achieve innovative breakthroughs with the help of supply chain finance. On the other hand, the market of traditional manufacturing enterprises is mature and the enterprise risk is relatively small, so it is necessary to consider whether to rely on supply chain finance to improve on the basis of traditional technology.

From the perspective of property right attribute, this paper further divides the whole sample into state-owned manufacturing enterprises and private manufacturing enterprises for sub sample regression. At present, China’s market environment is not very perfect, and hidden risks inside and outside the supply chain still exist. For risk avoidance and other reasons, there may be significant differences in the acquisition of innovative resources between state-owned enterprises and private enterprises in actual production and operation. Whether this difference caused by the property right attribute will affect the effect of Supply Chain Finance on enterprise innovation efficiency is also the focus of this paper.

#### (1) Inspection based on industrial attributes

According to the estimation results of models (1)–(3) in Table 7, supply chain financial services have a significant positive impact on the comprehensive innovation efficiency of the high-tech enterprise. For traditional manufacturing enterprises, according to models (4)–(6), the role of supply chain finance in promoting the innovation of traditional manufacturing enterprises is more reflected in the technological innovation of enterprises, and the influence coefficient is relatively higher than the empirical results of the whole sample.

**Table 7:** Results of sub-sample estimation based on industry attributes.

Sample attributes	High-tech industry sample						Traditional industry sample					
Model	(1)		(2)		(3)		(4)		(5)		(6)	
Explained Variables	CE		TE		OE		CE		TE		OE	
SCF	0.089*	0.144*	0.091*	0.083	0.094	0.138	0.112	0.093	0.163**	0.129*	0.078	0.172*
	(1.76)	(1.76)	(1.67)	(1.15)	(0.97)	(1.12)	(1.62)	(1.24)	(2.14)	(1.78)	(1.02)	(1.78)
TA		0.051*		0.037		0.039		0.035**		0.032		0.076**
		(1.94)		(1.55)		(1.26)		(2.07)		(1.27)		(2.50)
RNA		0.164***		0.290***		0.147		0.103**		0.167***		0.065
		(3.00)		(5.78)		(1.48)		(2.14)		(2.98)		(0.88)
AGE		-0.010**		0.023***		-0.055***		-0.008*		0.032***		-0.053***
		(-2.19)		(4.93)		(-6.19)		(-1.98)		(5.75)		(-7.15)
EPS		0.115		0.029		-0.036		0.303**		0.412**		0.329*
		(1.45)		(0.38)		(-0.21)		(2.07)		(2.61)		(1.86)
EM		-0.012		0.020		-0.012		0.023		0.026		-0.028
		(-0.36)		(0.71)		(-0.33)		(1.05)		(1.05)		(-1.20)
Constant	0.095***	-0.964*	0.181***	-0.696	0.460***	-0.180	0.101***	-0.700**	0.216***	-0.677	0.389***	-1.068*
	(14.47)	(-1.78)	(28.59)	(-1.45)	(28.23)	(-0.29)	(11.18)	(-2.03)	(16.48)	(-1.30)	(22.42)	(-1.70)
Number of samples	760						575					
Individual effect	Control											
Time effect	Control											

Note:

*, **, *** correspond to the significance level of 10%, 5%, and 1% respectively

Combined with the control variables, the possible explanation is that compared with traditional industries, high-tech industries need a lot of funds to support innovation and improvement due to the particularity of their own industries. Therefore, both the company size and the return level of enterprise assets have a significant positive effect on the comprehensive innovation efficiency of enterprises. The innovation of high-tech enterprises is relatively difficult, and it still takes time for the market to accept new products. In order to deal with market risks, high-tech enterprises need to consider multiple aspects from the perspectives of organization management and technological breakthrough when they obtain the financial support of supply chain to carry out innovation activities, which is finally reflected in the significant improvement of Supply Chain Finance on the comprehensive innovation efficiency of enterprises. However, the development mode of enterprises in traditional industries is mature, with sufficient information exchange and relatively high product recognition, which may make it easier to obtain financial support from the supply chain. Moreover, due to the relatively mature market and controllable overall risk of traditional manufacturing enterprises, traditional manufacturing enterprises can better guide capital investment into basic technology R & D through supply chain finance. The final performance is the significant improvement of Supply Chain Finance on the efficiency of enterprise technological innovation.

#### (2) Inspection based on property right attribute

Table 8 reports the results of separately estimating the sample into two types: state-owned enterprises and private enterprises according to the property right attribute. The estimation results of models (1)–(3) show that supply chain financial services have a significant negative impact on the organizational innovation efficiency of state-owned enterprises, and after the introduction of control variables, supply chain finance also has a negative impact on the comprehensive innovation efficiency of enterprises. The estimation results of models (4)–(6) show that for private enterprises, whether control variables are added or not, supply chain finance can significantly promote the comprehensive innovation efficiency, technological innovation efficiency and organizational innovation efficiency of enterprises.

**Table 8:** Results of sub-sample estimation based on property rights attributes.

Sample attributes	State-owned enterprise sample						Private enterprise sample					
Model	(1)		(2)		(3)		(4)		(5)		(6)	
Explained Variables	CE		TE		OE		CE		TE		OE	
SCF	-0.119	-0.275*	0.076	-0.183	-0.882**	-0.980***	0.107**	0.130**	0.125***	0.098*	0.118*	0.190**
	(-0.94)	(-2.05)	(0.66)	(-1.42)	(-3.01)	(-4.34)	(2.56)	(2.38)	(2.66)	(1.92)	(1.93)	(2.41)
TA		-0.004		0.033		0.033		0.047***		0.032*		0.059**
		(-0.08)		(0.63)		(0.41)		(2.76)		(1.83)		(2.57)
RNA		-0.021		0.178**		-0.378**		0.131***		0.232***		0.109*
		(-0.25)		(2.40)		(-2.26)		(3.55)		(5.84)		(1.75)
AGE		0.003		0.023***		-0.034		-0.010***		0.026***		-0.056***
		(0.39)		(3.26)		(-1.60)		(-3.27)		(7.13)		(-9.19)
EPS		0.144		-0.015		0.350		0.223**		0.224**		0.112
		(1.05)		(-0.10)		(1.39)		(2.43)		(2.38)		(0.84)
EM		0.080		0.111		0.081		0.005		0.024		-0.023
		(1.02)		(1.66)		(1.09)		(0.22)		(1.21)		(-1.04)
Constant	0.095***	0.028	0.150***	-0.757	0.536***	-0.215	0.098***	-0.904***	0.199***	-0.641*	0.425***	-0.629
	(9.52)	(0.03)	(9.96)	(-0.73)	(14.49)	(-0.13)	(17.95)	(-2.61)	(29.02)	(-1.77)	(34.23)	(-1.36)
Number of samples	760						575					
Individual effect	Control											
Time effect	Control											

Note:

*, **, *** correspond to the significance level of 10%, 5%, and 1% respectively

The possible explanation is: on the one hand, most of the small and medium-sized state-owned manufacturing enterprises have a dependency relationship or parent-child relationship with large enterprises through capital. Relying on the market advantages and strong capital advantages of large state-owned enterprises, they are easier to be recognized by the market when the external environment of the supply chain is imperfect. Therefore, in terms of access to innovation resources, small and medium-sized state-owned manufacturing enterprises have inherent advantages. Therefore, when state-owned manufacturing enterprises carry out innovation activities, supply chain financial funds are likely to be only a supplement and can not play an obvious role in promoting.

On the other hand, many state-owned enterprises obtain funds from the parent company and other institutions through related party transactions and then provide funds to the supply chain. With the increase of capital circulation in the supply chain, smes in the state-owned manufacturing industry, as a channel of capital circulation, may focus their business on financial services and neglect their own innovation and development. However, if financial risks occur to enterprises in the supply chain, it is easy to affect state-owned manufacturing smes through supply chain financial services, which is finally reflected in the empirical study as the negative impact of supply chain finance on the organizational innovation efficiency of state-owned enterprises.

For small and medium-sized private manufacturing enterprises, they often face the competitive pressure inside and outside the supply chain. In order to develop better, they often need to actively innovate in technology and management to obtain competitive advantage. Reflected in the control variables, enterprises with larger scale, stronger profitability and sustainable development ability also have higher innovation efficiency. Supply chain financial services provide reliable help for enterprises to solve the problem of insufficient supply of innovation factors dominated by financing constraints, which is reflected in the significant positive impact of supply chain financial services on the comprehensive innovation efficiency, technological innovation efficiency and organizational innovation efficiency of enterprises.

#### (3) Inspection based on dual attributes of industrial property rights

The regression results in Tables 9 and 10 show the impact of Supply Chain Finance on enterprise innovation efficiency under different property rights and industrial backgrounds. It can be seen from Table 9 that for private enterprises, supply chain financial services have a significant positive impact on the comprehensive innovation efficiency of high-tech enterprises, while for traditional manufacturing enterprises, it is more reflected in the impact on the technological innovation efficiency of enterprises. It is worth noting that the impact coefficient of Supply Chain Finance on the innovation efficiency of the two types of enterprises is also the highest in all samples. It can be seen from Table 10 that before the introduction of control variables, supply chain finance has a significant negative impact on the comprehensive innovation efficiency and organizational innovation efficiency of state-owned high-tech enterprises. After the introduction of control variables, this negative impact is also reflected in the comprehensive innovation efficiency and technological innovation efficiency of state-owned traditional manufacturing enterprises.

**Table 9:** The results of the sub-sample estimation results of private enterprises based on industrial attributes.

Sample attributes	Sample of private high-tech enterprises						Samples of private traditional enterprises					
Model	(1)		(2)		(3)		(4)		(5)		(6)	
Explained Variables	CE		TE		OE		CE		TE		OE	
SCF	0.102*	0.171*	0.098	0.092	0.149	0.227*	0.112	0.102	0.163**	0.137*	0.094	0.185*
	(1.84)	(1.77)	(1.61)	(1.06)	(1.48)	(1.72)	(1.60)	(1.36)	(2.07)	(1.87)	(1.23)	(1.90)
TA		0.054**		0.036		0.049		0.039**		0.033		0.081**
		(1.99)		(1.51)		(1.53)		(2.21)		(1.29)		(2.56)
RNA		0.177***		0.302***		0.178*		0.101**		0.159***		0.071
		(3.20)		(5.89)		(1.91)		(2.04)		(2.77)		(0.95)
AGE		-0.010**		0.023***		-0.058***		-0.009**		0.032***		-0.054***
		(-2.31)		(4.71)		(-6.25)		(-2.11)		(5.65)		(-7.03)
EPS		0.127		0.046		-0.035		0.312*		0.432**		0.311
		(1.48)		(0.55)		(-0.19)		(1.87)		(2.41)		(1.56)
EM		-0.018		0.017		-0.029		0.023		0.025		-0.023
		(-0.45)		(0.52)		(-0.67)		(1.02)		(0.96)		(-0.99)
Constant	0.095***	-1.005*	0.182***	-0.693	0.457***	-0.365	0.101***	-0.784**	0.221***	-0.691	0.379***	-1.175*
	(13.91)	(-1.84)	(27.30)	(-1.42)	(27.00)	(-0.57)	(10.50)	(-2.16)	(15.45)	(-1.30)	(20.95)	(-1.81)
Number of samples	735						530					
Individual effect	Control											
Time effect	Control											

Note:

*, **, *** correspond to the significance level of 10%, 5%, and 1% respectively

**Table 10:** State-owned enterprises’ sub-sample estimation results based on industrial attributes.

Sample attributes	Sample of state-owned high-tech enterprises						Sample of State-owned Traditional Enterprises					
Model	(1)		(2)		(3)		(4)		(5)		(6)	
Explained Variables	CE		TE		OE		CE		TE		OE	
SCF	-0.210***	-0.259***	-0.020	-0.156**	-1.394***	-1.391***	-0.061	-1.013***	0.126	-0.867***	-0.528	-0.845
	(-10.26)	(-6.90)	(-0.34)	(-4.14)	(-9.08)	(-6.09)	(-0.22)	(-5.63)	(0.52)	(-5.88)	(-1.06)	(-1.37)
TA		-0.031**		0.013		-0.080		-0.024		0.011		0.082
		(-3.21)		(0.69)		(-0.76)		(-0.52)		(0.19)		(0.89)
RNA		-0.055		0.141**		-0.349		0.026		0.321*		-0.482
		(-1.25)		(4.14)		(-2.09)		(0.15)		(2.09)		(-1.69)
AGE		0.025**		0.037**		0.024		0.009		0.029**		-0.041
		(3.93)		(3.67)		(1.15)		(0.68)		(3.29)		(-1.34)
EPS		-0.153**		-0.469***		-0.166		0.329		0.230		0.591
		(-2.89)		(-11.16)		(-0.51)		(1.42)		(1.00)		(1.75)
EM		-0.138		-0.047		-0.377		0.233**		0.250***		0.041
		(-1.91)		(-0.67)		(-1.31)		(2.63)		(3.74)		(0.33)
Constant	0.071***	0.919***	0.124***	-0.045	0.570***	2.838	0.111***	0.253	0.167***	-0.504	0.527***	-1.196
	(7.19)	(7.11)	(4.84)	(-0.10)	(13.29)	(1.53)	(5.86)	(0.26)	(8.77)	(-0.43)	(10.35)	(-0.63)
Number of samples	25						45					
Individual effect	Control											
Time effect	Control											

Note:

*, **, *** correspond to the significance level of 10%, 5%, and 1% respectively

The possible explanation is as follows: as a private enterprise, supply chain financial funds are an important supplementary source of funds. Under the condition of limited resources, enterprises first need to consider how to flow factors to the place that can best promote the development of enterprises. For high-tech enterprises, the market may not be fully mature. In the face of strong competitive pressure, in order to better obtain advantages in the new market, enterprises need not only make breakthroughs in technology, but also make timely adjustments in organization and management. Therefore, supply chain finance significantly promotes the comprehensive innovation efficiency of private high-tech enterprises. From the sample of private traditional enterprises, due to the relative stability of the market, the capital, technology and other innovation elements flowing to enterprises relying on the supply chain may be more used for the innovation and improvement of existing technologies, and new products are easier to be recognized when flowing through the supply chain, which can realize the benefits of rapid return of funds and achievement conversion, Therefore, the empirical results reflect the significant positive impact of Supply Chain Finance on the technological innovation efficiency of private traditional manufacturing enterprises.

Corresponding to private enterprises, it can be seen from Table 10 that under the background of state-owned enterprises, whether it is the sample of traditional manufacturing enterprises or the sample of high-tech enterprises, supply chain finance basically has a significant negative impact on all kinds of innovation efficiency of enterprises, and this negative impact is most obvious in the organizational innovation efficiency of state-owned high-tech manufacturing enterprises. In view of this characteristic of state-owned enterprises, the explanation of this paper is the same as the above, that is, state-owned manufacturing enterprises may be based on the special identity of state-owned enterprises. When the external market environment is imperfect, innovation factors are more likely to accumulate in state-owned manufacturing enterprises, which may lead to weak innovation will of enterprises. In addition, by relying on large state-owned enterprises, small and medium-sized state-owned enterprises can act as the provider of supply chain financial services to earn remuneration. Therefore, when the market demand for supply chain financial services increases, state-owned enterprises may use more funds in financial services and ignore their own enterprise innovation and development.

## 6 Conclusion

China’s supply chain financial services have developed rapidly in recent years, and whether China’s manufacturing small and medium-sized enterprises can rely on supply chain finance to better help enterprises improve innovation efficiency has become the focus of this paper. In terms of theoretical mechanism, supply chain financial services can directly promote the innovation efficiency of enterprises, or promote the innovation efficiency of small and medium-sized enterprises by spillover of innovation efficiency of core enterprises and optimizing the competition and cooperation relationship in the supply chain financial system. However, when there are risk factors inside the supply chain, the external environment is unstable or adverse selection occurs, it may also inhibit the innovation efficiency of enterprises.

By selecting the data of 267 gem manufacturing enterprises from 2015 to 2019 for empirical analysis, the results show that: Supply chain financial services have a strong positive impact on the comprehensive innovation efficiency, technological innovation efficiency and organizational innovation efficiency of small and medium-sized manufacturing enterprises. Further, from the perspective of industry, supply chain financial services have a significant positive impact on the comprehensive innovation efficiency of high-tech enterprises and the technological innovation efficiency of small and medium-sized enterprises in traditional manufacturing industry. Based on the property right attribute analysis, supply chain finance has a significant negative impact on the organizational innovation efficiency of small and medium-sized state-owned manufacturing enterprises. It has a statistically significant positive impact on the comprehensive innovation efficiency, technological innovation efficiency and organizational innovation efficiency of small and medium-sized private manufacturing enterprises. Based on the dual attributes of industrial property rights, supply chain finance has the most significant improvement on the technological innovation efficiency of private traditional enterprises, but it has a significant inhibitory effect on the organizational innovation efficiency of state-owned high-tech enterprises.

The limitation of this paper is that it has not conducted empirical tests on some parts of the theoretical mechanism, thus its persuasiveness maybe is somewhat insufficient. The next research direction is to test the assumptions in the theoretical mechanism. In addition, the selected sample is a sample of Chinese enterprises, which requires more data to be analyzed.

Based on the research results, combined with the current development trend of supply chain financial services, this paper puts forward the following three enlightenment suggestions:

Firstly, the development of supply chain financial services cannot ignore the support for traditional manufacturing. In essence, supply chain financial services rely on the industrial chain, and the health and stability of the industrial chain is the foundation for the development of supply chain financial services. In recent years, the Chinese economy has been moving towards high-quality development, and one of the main characteristics of the high-quality development of the manufacturing industry is the gradual optimization of the industrial chain. Although the optimization of the industrial chain must be achieved through the development of high-tech industries, China is a large manufacturing country with a large proportion of traditional industries. The potential and demand for the upgrading and transformation of traditional manufacturing are also huge, and the upgrading and transformation of traditional industries is also an important link in the optimization of the industrial chain.

Secondly, the development of supply chain financial services should be inclined to private enterprises and stimulate the innovation potential of private enterprises. Private enterprises have become an important force in China’s economic development and the main force of China’s innovation and development. Although from the perspective of China’s economic development system, private enterprises still face many development constraints, the vitality and innovation of their development need the active participation of supply chain financial services. Their demand for supply chain financial services should bring driving force to the development of supply chain financial services rather than avoidance.

Furthermore, We will improve relevant laws and regulations, establish and improve relevant institutional arrangements, and encourage state-owned enterprises to participate in market competition. At present, due to the lack of corresponding laws in some areas, some institutional arrangements are relatively lagging behind. The development of supply chain finance often depends on the internal communication and negotiation of supply chain enterprises. For the consideration of credit risk, financial risk and other factors, without effective supervision, many core enterprises often choose state-owned manufacturing enterprises with relatively small wind direction when providing supply chain financial help, and raise the help requirements for private enterprises. Therefore, this paper proposes to strengthen the relevant legislation of supply chain finance, especially the relevant provisions on asset mortgage and asset disposal; Establish corresponding institutional arrangements, actively guide the development of supply chain finance, and effectively ensure the sound and healthy development of supply chain finance; Implement effective supervision and guide state-owned enterprises to actively participate in market competition, so as to prevent the excessive agglomeration of innovation resources to state-owned enterprises, resulting in the decline of the innovation efficiency of the overall supply chain.

## Supporting information

S1 Data (XLSX)
